# Supplementary material for: Asymmetry between Activation and Deactivation during a Transcriptional Pulse
Source: Cell Syst. 2017 Dec 27;5(6):646–653.e5. doi: 10.1016/j.cels.2017.10.013 (PMC5747351; doi:10.1016/j.cels.2017.10.013)
Supplement: Document S1. Figures S1–S4 and Tables S2 and S3 [file mmc1.pdf]

Cell Systems, Volume *5*

Supplemental Information

Asymmetry between Activation and Deactivation during a Transcriptional Pulse

Lee S.S. Dunham, Hiroshi Momiji, Claire V. Harper, Polly J. Downton, Kirsty Hey, Anne McNamara, Karen Featherstone, David G. Spiller, David A. Rand, Bärbel Finkenstädt, Michael R.H. White, and Julian R.E. Davis

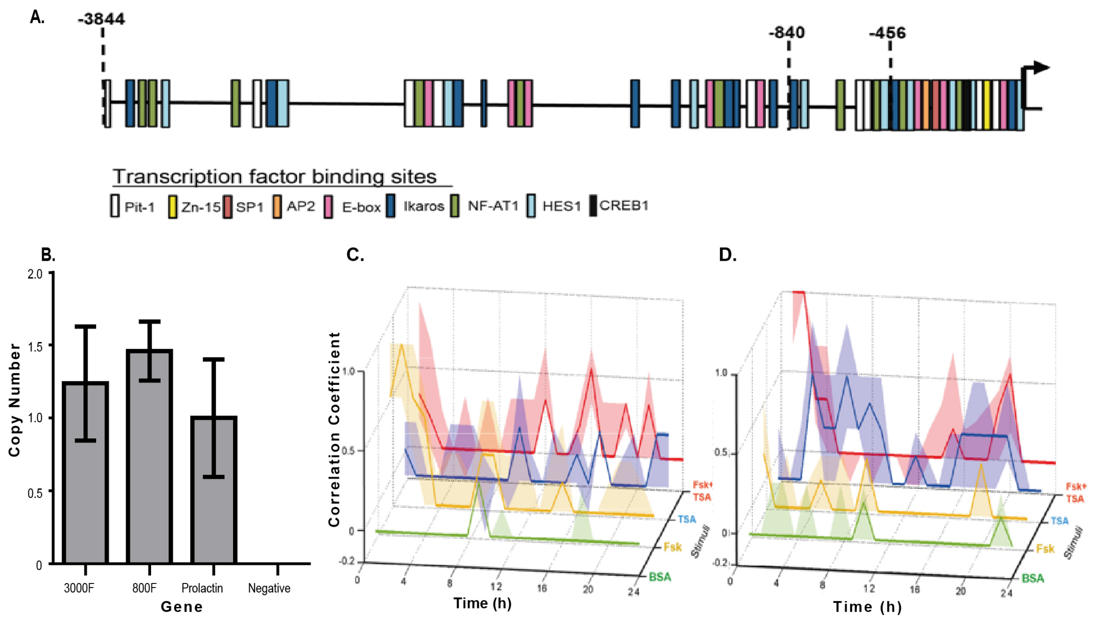

Figure S1 relating to Figure 1. A hGH promoter schematic of known transcription factor binding sites up to -456bp and bioinformatic estimated sites up to -3844bp. Binding site estimation performed with Transfac analysis weight limits: matrix = 0.9, core = 0.95. **B** Copy number validation of transformed GH3 cell lines, containing either the -840/+1bp or -3348/+1bp human growth hormone promoter driving the expression of the luciferase reporter gene. Each boxplot represents the mean copy number and standard deviation (*n* = *3*). **C-D** Cell-cell luminescence pattern correlation in response to stimulation. Correlation coefficients were calculated of 1h pooled luminescence profiles for each cell pair combination within a population for a 20h observation period. The median correlation coefficient (bold) and the associated 95% confidence interval (shaded) demonstrates the coordination in luminescence activity in unstimulated (BSA) conditions (green), forskolin (Fsk) (yellow), trichostatin A (TSA) (blue), and Fsk + TSA (red).

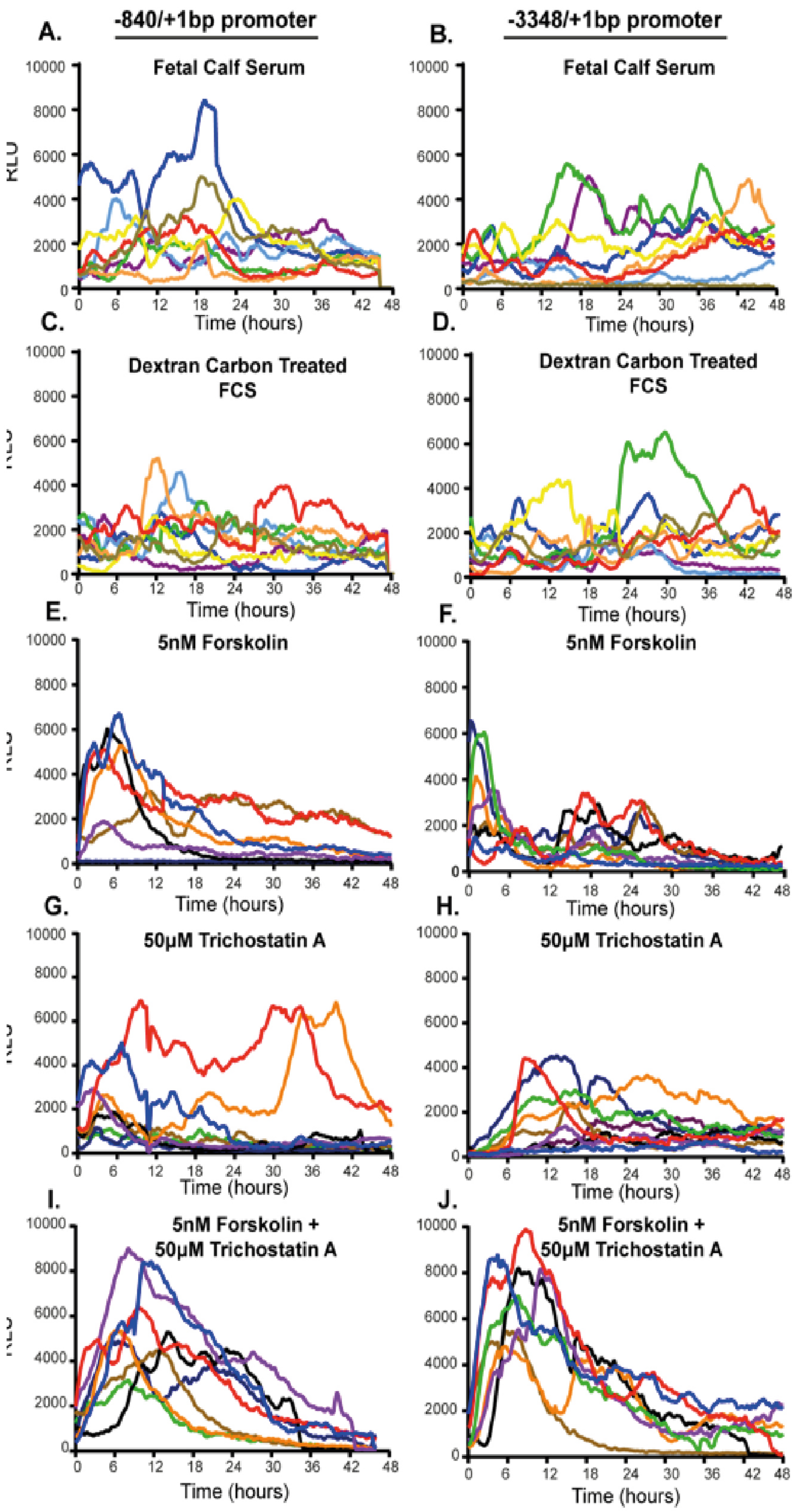

Figure S2 relating to Figure 1. A-J Representative single cell luminescence traces generated by -840/+1bp or -3348/+1bp human growth hormone promoter driven luciferase expression in GH3 cells, in response to serum and non-serum conditions, and stimulation with 5nM forskolin, 50μM trichostatin A, or a combination of both when maintained in BSA media. (-840/+1bp Fsk *n*=41, TSA *n*=33, Fsk+TSA *n*=46; -3348/+1bp Fsk *n*=59, TSA *n*=42, Fsk+TSA *n*=40).

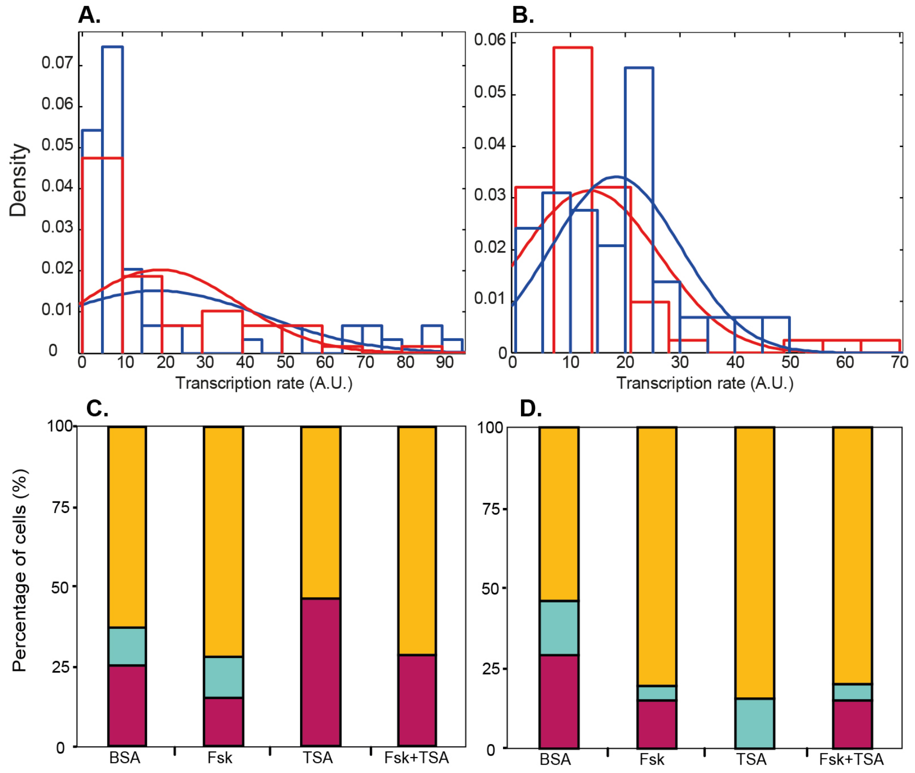

Figure S3 related to Figure 3. A-B Frequency distribution of stochastic switch model estimated transcription rates at the start (blue) and end (red) of the 48h observation period of the **A**. -840/+1bp and **B**. -3348/+1bp promoter-containing GH3 cells in BSA media. Kolmogorov-Smirnov test, $p>0.05$. **C-D** The percentage of cells in a population over a 48h period exhibiting either a binary, multi-step up, or multi-step down switch profile. When stimulated (Forskolin, trichostatin A, or combination) the percentage of cells containing either the **C**. hGH -840/+1bp or **D**. hGH -3348/+1bp promoter does not significantly change the switch profile they exhibit (ANOVA, $p > 0.05$).

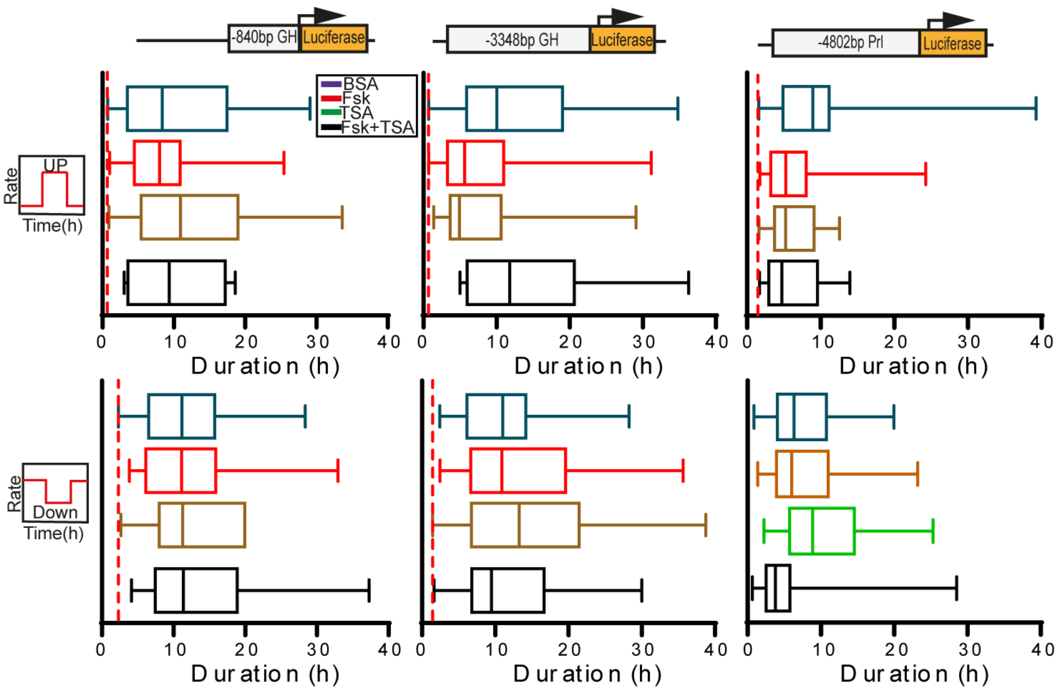

Figure S4 related to Figure 4. Boxplots demonstrating the median, interquartile range, and range of the stochastic switch model (SSM) estimated periods of transcription categorised as either Up or Down, depending upon the switch preceding each period for the -840/+1bp and -3348/+1bp hGH promoter constructs. Each boxplot represents the distribution of period durations in response to serum-starvation (BSA) (blue), forskolin (Fsk) (red), trichostatin A (TSA) (green), and a combination of Fsk and TSA (black).The dashed red line indicates the minimum estimated duration.

	BSA	Fsk	TSA	Fsk + TSA
Protein half-life	0.98 (±0.03)<br>**58.8m**	0.98 (±0.02)<br>**58.8m**	0.98 (±0.15)<br>**58.8m**	1.11 (±0.08)<br>**66.6m**
mRNA half-life	0.58 (±0.07)<br>**34.6m**	0.6 (±0.01)<br>**36m**	0.53(±0.01)<br>**31.8m**	0.61(±0.02)<br>**36m**

Table S2 related to Figure 2. Luciferase half-life used to determine protein and mRNA degradation rates following serum-starvation, or treatment with forskolin (Fsk) or trichostatin A (TSA). Half-life calculation (±SD) based on observed luminescence profile following treatment with cycloheximide (protein) or actinomycin D (mRNA half-life), and converted into minutes (**bold**).

		Promoter construct					
		**hGH -840/+1bp**		**hGH -3348/+1bp**		**hPrl -5kb**	
		Slope	*R^2*	*Slope*	*R^2*	*Slope*	*R^2*
**Consecutive switch directions**	*Up-Down*	-1.04 *(±0.04)*	*-0.95 *(±0.1)*	-1.08 *(±0.1)*	*0.99 *(±0.1)*	-0.96 *(±0.03)*	*0.94 *(±0.2)*
	Down-Down	1.76 *(±0.5)*	*0.60 *(±0.3)*	2.33 *(±0.9)*	*0.69 *(±0.1)*	1.92 *(±0.8)*	0.83 *(±0.1)*
	Up-Up	0.65 *(±1.7)*	-0.45 *(±0.9)*	-0.60 *(±0.3)*	0.39 *(±0.4)*	-0.33 *(±1.3)*	0.48 *(±0.4)*
	Down-Up	-0.73 *(±1.8)*	0.004 *(±1.9)*	0.15 *(±1.2)*	0.45 *(±0.7)*	0.26 *(±0.9)*	0.33 *(±0.5)*

Table S3 related to Figure 3. Mean (n=3) slope and R^2 values (±SD) calculated from the relationship between consecutive transcriptional switch amplitudes of -840/+1bp, -3348/+1bp human growth hormone and -5kb human prolactin promoters estimated by the stochastic switch model. *R^2>0.5.
