# Supplementary material for: Asymmetry between Activation and Deactivation during a Transcriptional Pulse
Source: Cell Syst. 2017 Dec 27;5(6):646–653.e5. doi: 10.1016/j.cels.2017.10.013 (PMC5747351; doi:10.1016/j.cels.2017.10.013)
Supplement: Document S2. Article plus Supplemental Information [file mmc3.pdf]

Cell Systems

Report

Asymmetry between Activation and Deactivation during a Transcriptional Pulse

Graphical Abstract

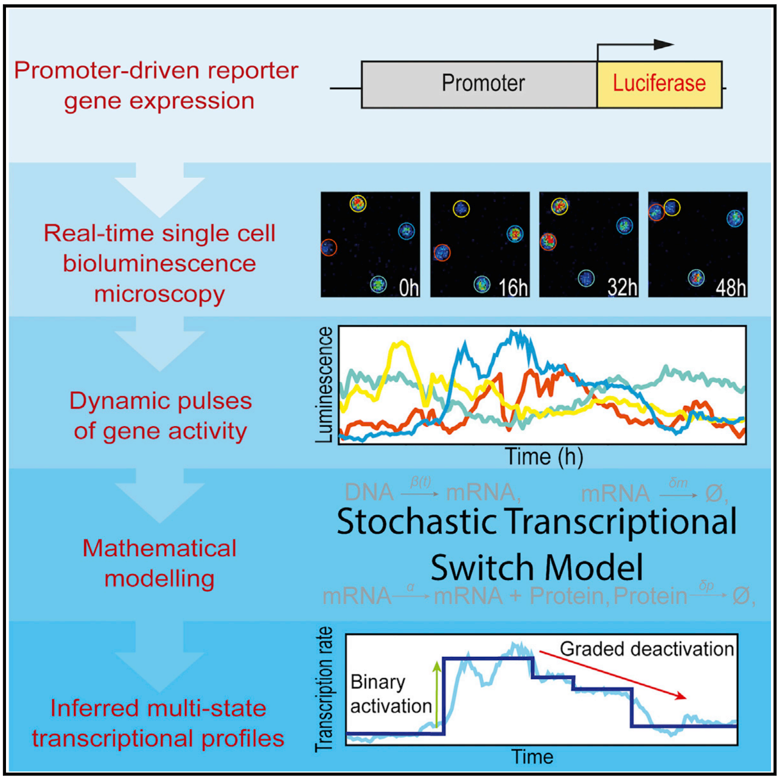

Authors

Lee S.S. Dunham, Hiroshi Momiji, Claire V. Harper, ..., Bärbel Finkenstädt, Michael R.H. White, Julian R.E. Davis

Correspondence

barbel.finkenstadt@warwick.ac.uk (B.F.), mike.white@manchester.ac.uk (M.R.H.W.), julian.davis@manchester.ac.uk (J.R.E.D.)

In Brief

Dunham et al. apply mathematical tools to single-cell imaging data to quantify gene expression dynamics, revealing a pattern of all-or-nothing activation and a graded deactivation in the regulation of transcriptional bursts.

Highlights

- Gene transcription switches between variable rates
- Single-cell microscopy and mathematical modeling quantifies switch dynamics
- We observe an asymmetry in the activation/deactivation of transcriptional bursts

CrossMark

Dunham et al., 2017, Cell Systems *5*, 646–653
December 27, 2017 © 2017 The Author(s). Published by Elsevier Inc.
https://doi.org/10.1016/j.cels.2017.10.013

CellPress

OPEN ACCESS
CellPress

Cell Systems
Report

Asymmetry between Activation and Deactivation during a Transcriptional Pulse

Lee S.S. Dunham,[1] Hiroshi Momiji,[2] Claire V. Harper,[3] Polly J. Downton,[3] Kirsty Hey,[4] Anne McNamara,[3] Karen Featherstone,[1] David G. Spiller,[3] David A. Rand,[2] Bärbel Finkenstädt,[4,*] Michael R.H. White,[3,*] and Julian R.E. Davis[1,5,*]

[1]Division of Endocrinology, Diabetes and Gastroenterology, School of Medical Sciences, Faculty of Biology, Medicine and Health, Manchester Academic Health Science Centre, University of Manchester, AV Hill Building, Manchester M13 9PT, UK
[2]Warwick Systems Biology Centre, University of Warwick, Coventry CV4, 7AL, UK
[3]Division of Cellular and Molecular Function, School of Biological Sciences, Faculty of Biology, Medicine and Health, Manchester Academic Health Science Centre, University of Manchester, Manchester M13 9PT, UK
[4]Department of Statistics, University of Warwick, Coventry CV4 7AL, UK
[5]Lead Contact
*Correspondence: barbel.finkenstadt@warwick.ac.uk (B.F.), mike.white@manchester.ac.uk (M.R.H.W.), julian.davis@manchester.ac.uk (J.R.E.D.)
https://doi.org/10.1016/j.cels.2017.10.013

SUMMARY

Transcription in eukaryotic cells occurs in gene-specific bursts or pulses of activity. Recent studies identified a spectrum of transcriptionally active "on-states," interspersed with periods of inactivity, but these "off-states" and the process of transcriptional deactivation are poorly understood. To examine what occurs during deactivation, we investigate the dynamics of switching between variable rates. We measured live single-cell expression of luciferase reporters from human growth hormone or human prolactin promoters in a pituitary cell line. Subsequently, we applied a statistical variable-rate model of transcription, validated by single-molecule FISH, to estimate switching between transcriptional rates. Under the assumption that transcription can switch to any rate at any time, we found that transcriptional activation occurs predominantly as a single switch, whereas deactivation occurs with graded, stepwise decreases in transcription rate. Experimentally altering cAMP signalling with forskolin or chromatin remodelling with histone deacetylase inhibitor modifies the duration of defined transcriptional states. Our findings reveal transcriptional activation and deactivation as mechanistically independent, asymmetrical processes.

INTRODUCTION

The expression of many genes has been shown to be highly dynamic and heterogeneous in individual living cells (Raj et al., 2006; Harper et al., 2011). Transcription is an inherently noisy process contributing to intercellular variation, where low molecular numbers amplify this variation (Becskei et al., 2005). An individual cell is subject to both *intrinsic* noise, which arises from probabilistic interactions between low numbers of intracellular molecules, and *extrinsic* noise, which is due to more global variations in cell states (Elowitz et al., 2002; Carey et al., 2013; Dadiani et al., 2013). Production of mRNA transcripts occurs in gene-specific bursts, reliant on promoter architecture (Zoller et al., 2015; Suter et al., 2011; Hocine et al., 2015), chromatin status (Bintu et al., 2016; Noordermeer et al., 2011), and exogenous stimuli (Harper et al., 2011; Molina et al., 2013; Featherstone et al., 2016). These bursts of activity are separated by intermittent "off" periods of transcriptional inactivity (Chubb et al., 2006; Zenklusen et al., 2008; Noordermeer et al., 2011). It has been suggested that this inactive phase comprises multiple independent states (Zoller et al., 2015). This phase includes a refractory period, during which a new round of transcriptional activation cannot be initiated (Harper et al., 2011; Suter et al., 2011). The rate-limiting duration of this state is assumed to be a result of a combination of processes including signaling, chromatin remodeling, transcription factor complex formation, and the recruitment and activation of RNA polymerase II (Larson et al., 2011; Sorre et al., 2014: Bintu et al., 2016).

Quantitative analysis of optical reporter genes, such as the firefly luciferase gene, provides real-time measurements of transcription dynamics from individual living cells. This enables the development of mathematical models and the formulation of subsequent hypotheses regarding the nature of bursts of promoter activity in a noisy molecular system. Previously applied "random telegraph" models assumed that transcription operated as a binary function, identifying defined on/off states of activity (Raj and Oudenaarden, 2009; Harper et al., 2011; Suter et al., 2011). However, observation of transcription profiles from large numbers of individual cells in either a basal or stimulated state suggested that variable rates may in fact occur (Harper et al., 2011; Molina et al., 2013; Corrigan and Chubb, 2014), and therefore an on/off model is too simplistic to describe dynamic transcription. While several studies have applied multistate models of transcription, many categorize transcriptional phases into only a few discrete states (Neuert et al., 2013; Zhang and Zhou, 2014; Zoller et al., 2015; Bintu et al., 2016). Hey et al. (2015) developed a stochastic switch model (SSM) assuming

This is an open access article under the CC BY license (http://creativecommons.org/licenses/by/4.0/).

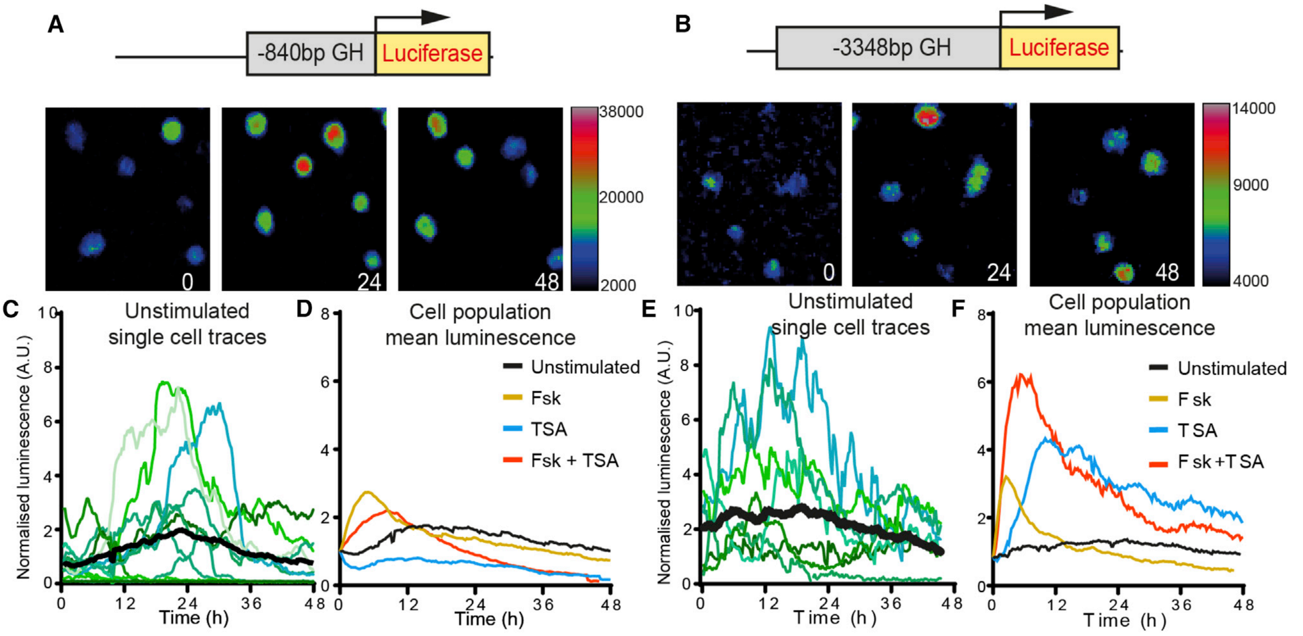

Figure 1. Heterogeneous Promoter-Specific Activity in Single Cells

(A and B) Example images of single-cell bioluminescence in GH3 pituitary cells expressing either an −840/+1 bp (A) or −3,348/+1 bp (B) *hGH*-luciferase transgene in serum-starved conditions (BSA) for 0–48 hr.

(C and E) Real-time luminescence plots demonstrate heterogeneous expression dynamics (shades of green/blue) but an overall stable population output (black).

(D and F) Mean population luminescence following stimulation shows promoter-specific responses. While the luminescence of both promoters is tripled within 4 hr upon cAMP stimulation with forskolin (Fsk), only the larger −3,348/+1 bp construct responds with increased output following HDAC inhibition by trichostatin A (TSA). The combination of Fsk and TSA has a synergistic effect, further increasing −3,348/+1 bp luminescence production. (−840/+1 bp: BSA, n=75; Fsk, n = 41; TSA, n=33; Fsk + TSA, n=46; −3,348/+1 bp: BSA, n = 97; Fsk, n = 59; TSA, n = 42; Fsk + TSA, n = 40).

that transcription could occur at any rate, and could switch in any direction at any time. When applied to the transcription dynamics of the human prolactin (hPrl) gene within the pituitary gland, the model provided a more graded or analog view of transcription (Featherstone et al., 2016). By not limiting the process to discrete transcriptional on-states, the model also provides a more quantitative insight into dynamic and complex transcriptional states, supporting recent work suggesting the occurrence of transcription along a continuum of rates (Corrigan et al., 2016; Sepúlveda et al., 2016).

Previous single-cell bioluminescence analysis of luciferase activity driven by a short human growth hormone (*hGH*) proximal promoter (−496/+1 bp) has highlighted the pulsatile dynamics of this gene (Norris et al., 2003). The cell-type-specific expression of this pituitary hormone is controlled by an extensive sequence that regulates chromatin remodeling and proximal promoter access (Ho et al., 2002, 2011, 2015; Shewchuk et al., 2002, 2006; Bodner et al., 1988; Lipkin et al., 1993; Cohen et al., 1999; Alonso et al., 1998). We have taken advantage of the pulsatile nature of hGH gene expression to assess the influence of different types of regulatory element on gene transcription dynamics. Furthermore, we compared these dynamics with those of the hPrl gene, an independently regulated pituitary hormone gene (Niall et al., 1971). The statistical modeling approach was validated by single-molecule fluorescence *in situ* hybridization (smFISH), quantitatively confirming the modeled estimates of the distribution of numbers of mRNA molecules. These data indicate that for each promoter there is an all-or-nothing "on-switch" in transcriptional activation, whereas transcriptional inactivation involves a decaying series of "off-switches." Our data suggest mechanistic hypotheses for the way in which promoters may fully engage with transcriptional machinery and then may switch off through a series of distinct states.

RESULTS

Promoter Structure and Associated Chromatin Remodeling Confer Expression Dynamics

To test the importance of promoter complexity on the pattern of pulsatile expression, we created two reporter cell lines by stably transfecting one copy of a −840/+1 bp or −3,348/+1 bp *hGH* proximal promoter-*luciferase* construct into the GH-expressing GH3 rat pituitary cell line (Figures 1A, 1B, S1A, and S1B). Both stable transfectant cell lines exhibited dynamic and heterogeneous luminescence activity in the presence or absence of serum or stimuli (Figures 1A–1F and S2). The luminescence produced by either *hGH* construct was approximately tripled following stimulation of the cyclic AMP (cAMP) signaling pathway with forskolin (Fsk) (Figures 1D and 1F). However, while the −840/+1 bp promoter showed no response to the inhibition of histone deacetylase (HDAC) by trichostatin A (TSA), the larger −3,348/+1 bp promoter greatly increased luminescence output. The combination of Fsk with TSA produced a synergistic response, further increasing population luminescence. The

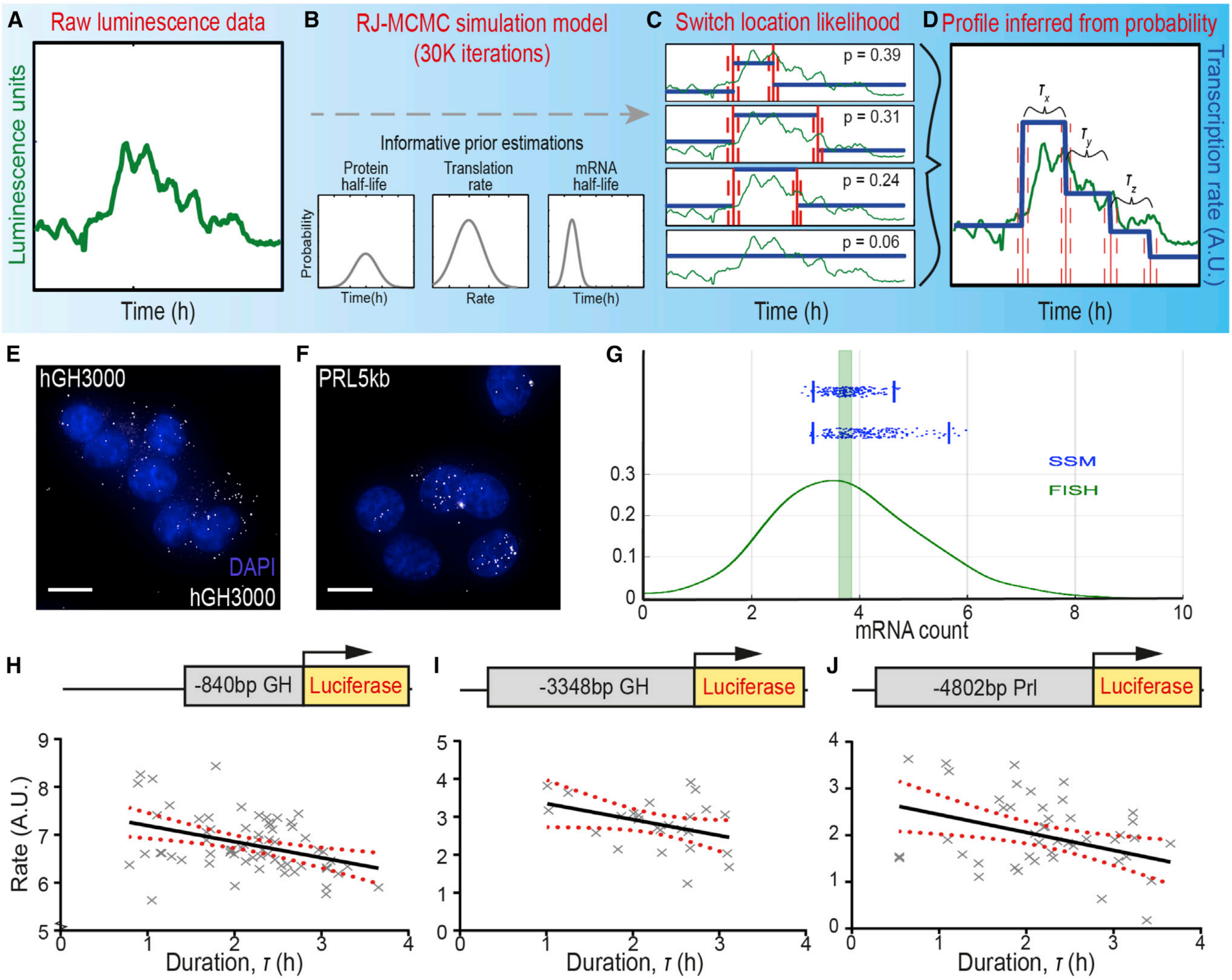

Figure 2. Estimation of Variable Transcription States Using a Stochastic Switch Model

(A–D) Schematic representation of the stochastic switch model (SSM) estimating transcription rate switches from single-cell bioluminescence traces (A) (green). (B) A reversible jump Markov chain Monte Carlo (RJ-MCMC) algorithm utilized known protein and mRNA half-life distributions (Table S2) and estimated translation rate distributions to back-calculate from observed luminescence to unobserved transcription rate switch events. (C) For a given luminescence profile (green), 30,000 iterations of the model estimated the location (red) and credible intervals (red dashes) of changes in transcription rate (blue), estimating several switch profiles with varying probability. (D) The probability of different switch profiles inferred a single likely transcriptional switch profile with quantified transcription phase duration ($\tau_{x,\ y,\ z}$…).

(E and F) smFISH analysis of luciferase mRNA transcript level in unstimulated reporter cell lines. Images are maximum-intensity projections of deconvolved z stacks, with luciferase transcript staining in white and DAPI counterstaining in blue. Scale bars, 10 μm.

(G) Numbers of mRNA molecules at t = 4 hr in unstimulated hGH3000 cells counted in smFISH experiments (green) and two separate estimates (n = 200 iterations) of mRNA molecules using estimated posterior distributions of the parameters of mRNA equation from the SSM (blue). Vertical bar represents 95% normal confidence interval of the mean over 549 cells.

(H–J) Plotting the ln-transformed SSM-estimated rate and duration of individual transcription phases (τ) (gray crosses) identified a significant inverse correlation (black line, with 95% confidence interval indicated by red dashed lines), with the duration of higher transcriptional rates being shorter than lower rates (Pearson's r, $p < 0.03$).

correlation of luminescence patterns between individual cells (Figures S1C and S1D) suggests that this synergistic increase was not due to increased activity of an already active subpopulation but to a reduction in cell-cell heterogeneity. We and others (Harper et al., 2011; Suter et al., 2011) have proposed that the "off-state" involves a refractory period that originates from a period of chromatin remodeling, during which a new round of transcription cannot be initiated. For the larger promoter this chromatin-remodeling step may promote population heterogeneity, and therefore protect against noisy activation.

Estimation of Transcriptional States Using a Stochastic Switch Model Suggests a Continuum of Rates

An SSM was previously developed and used here for inference on switches in transcription rate from the observed reporter gene activity (Figure 2A) through the fitting of various parameter

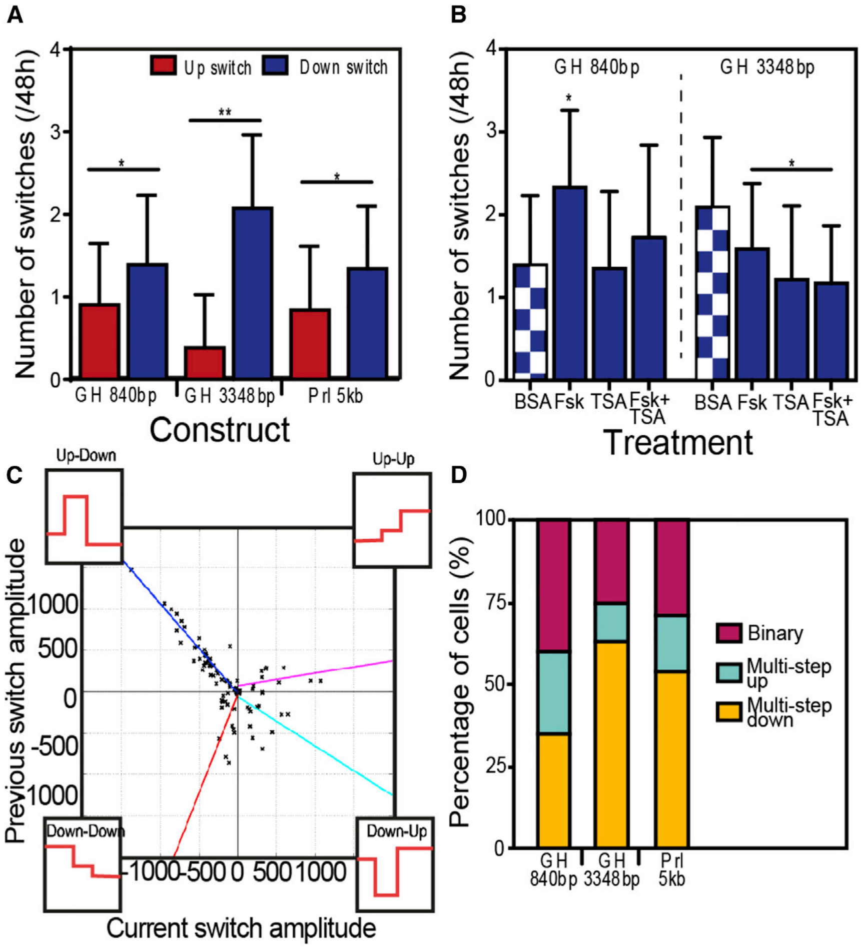

Figure 3. Quantification of Switch Characteristics

(A) The mean number and SEM of estimated switches increasing (Up) or decreasing (Down) transcription rate when cell lines are serum starved for a 48-hr time course. All promoters, including the 5-kb Prl promoter, produce significantly more Down switches (ANOVA *p < 0.05, **p < 0.001).

(B) The effect of stimulation on the mean number and SEM of Down switches produced by the *hGH*-luciferase constructs, compared with the serum-starved (BSA) response (chequered) replicated from (A). The inhibition of an HDAC chromatin-remodeling mechanism by TSA treatment significantly reduces the number of Down switches produced by the −3,348/+1 bp construct (ANOVA *p < 0.05).

(C) By comparing the amplitude of a current transcription rate switch with the amplitude of a previous switch (black crosses), we identified four switch-pair scenarios (Up-Down, Down-Down, Down-Up, and Up-Up). The amplitudes of consecutive Up-Down and Down-Down switch pairs strongly correlate (respective colored lines represent correlation calculated in Figure S4), suggesting a mechanistic memory controlling the activation and sequential deactivation of transcription (−840/+1 bp, n=76; −3,348/+1 bp, n=100; Prl, n = 71; from three experimental repeats).

(D) The percentage of cells in a population exhibiting either a binary or graded transcriptional profile. Cell profiles are characterized by the estimation of a binary-like switch, a graded increase, or a graded decrease within a 48-hr time course. Over 50% of each cell line exhibited graded regulation of transcriptional activity.

prior distributions (Figure 2B) (Hey et al., 2015). Through 30,000 iterations of this reverse jump-Markov chain Monte Carlo-based algorithm, the locations of significant changes in transcription rate were identified. These estimations produced several potential switch profiles for each single-cell luminescence trace, each with varying probability (Figure 2C). These probable scenarios inform the generation of a single likely transcription profile (Figure 2D).

The SSM was evaluated using smFISH (Figures 2E and 2F) as an independent and directly quantitative assay to measure to distribution of mRNA molecule numbers. This showed close agreement between observed and modeled mRNA numbers (Figure 2G). Starting with the assumption that any transcription rate is possible, and that switching between variable rates is independent of the previous rate, we observed a significant inverse relationship between the rate and duration (τ) of a transcriptional phase when fitted to the single-cell luminescence data produced by each *hGH*- and *hPrl*-luciferase promoter constructs (Figures 2H–2J). High rates of transcriptional activity lasted for the shortest periods of time, while low rates of transcription could be maintained for longer. The continuous distribution of rates as modeled by the SSM approach supported the view that rather than this being a discrete binary on/off system, transcription occurs along a spectrum of possible rates (Molina et al., 2013; Zhang and Zhou, 2014; Featherstone et al., 2016; Corrigan et al., 2016; McNamara et al., 2016).

Asymmetrical Transcriptional Control

Unlike previous binary models of transcriptional activity, the SSM used here estimates a switch in rate in any direction, independent of any previous switch direction or rate. Using such assumptions, we observed an unequal number of rate-increasing and rate-decreasing switches, with approximately twice as many “down-switches” identified per cell for all *hGH*- and *hPrl* promoter constructs throughout a 48-hr period (Figure 3A). Despite this asymmetry in the number of these deactivating switches, the overall transcription rate across the entire cell population was maintained (Figures S3A and S3B), indicating a constant and stable population output. Therefore the greater number of down-switches was not due to a general systematic reduction in transcriptional activity throughout the experimental period, but instead indicates the nature of the underlying process. A slight increase in the number of deactivating switches is observed following Fsk stimulation of the −840/+1 bp fragment (Figure 3B), but the number of down-switches remains unaffected by TSA treatment. A significant decrease in the number of these deactivating steps is seen of the −3,348/+1 bp promoter when HDAC is inhibited, reducing the number of switches by half.

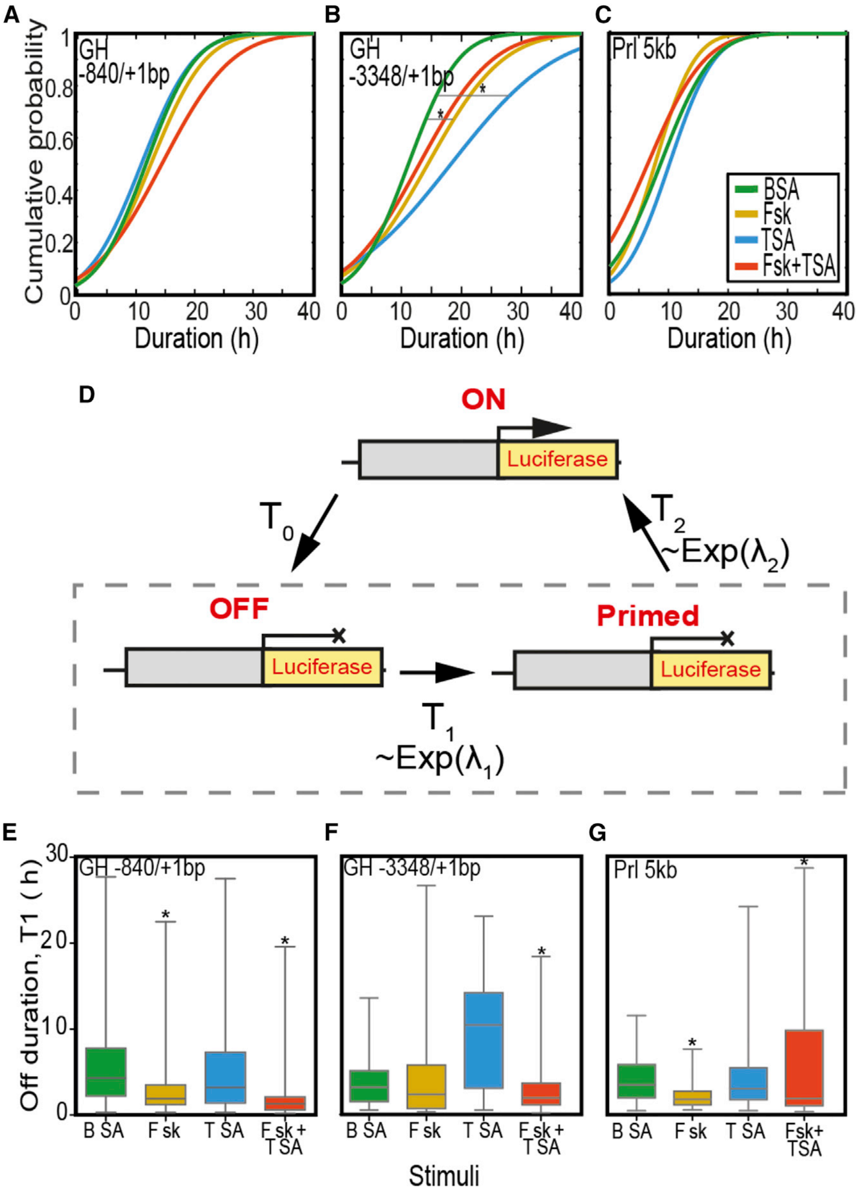

Figure 4. Identification and Quantification of Low Transcriptional States

(A–C) The cumulative distribution of the duration of decreased periods of transcription, as estimated by the SSM. HDAC inhibition (TSA) variably effects the inactivity of the different promoter fragments, with the most significant effect observed in the increased probability of a longer duration generated by the extended promoters (Kolmogorov-Smirnov, *p < 0.05).

(D) A three-state model was produced to quantify these inactive periods as two independent phases, OFF and Primed.

(E–G) The durations shown in (A) to (C) were applied to the iterative three-state model (D) identifying the median, interquartile range (box), and full range (whiskers), of the duration of the refractory (Off) period. The most notable modification to this refractory period is following HDAC inhibition (TSA), reducing the refractory period of the GH −3,348/+1 bp promoter (F), but not of the smaller GH −840/+1 bp promoter (E) (Mann-Whitney, *p < 0.001).

Figure 3C plots the amplitude of two consecutive switches allowing for the identification of any consistent switch patterns. We identify predictable patterns for each scenario when a Down switch is the secondary switch, with Up-Down and Down-Down switch amplitudes being highly correlated (Table S3). This predictability suggests a system memory controlling the transcription-deactivating mechanism. Over 50% of each cell line consistently exhibits a graded pattern of transcriptional switching, with more cells containing the larger constructs producing the asymmetrical graded deactivation pattern (Figures 3D, S3C, and S3D).

While previous research has suggested a transcriptional pulse to be either binary or graded (Biggar and Crabtree, 2001; Giorgetti et al., 2010; Bintu et al., 2016; Ochab-Marcinek and Tabaka, 2015), we propose that a single transcriptional burst is predominantly a combination of an all-or-nothing activation, followed by a graded reduction, tuning transcriptional output along a continuum to the post-stimulatory environment.

Quantifying Mechanistic Influences on Transcription Phase Duration

Noting the characteristic differences between the regulation of transcriptional activation and deactivation (Figures 2 and 3), we investigated a modification to the duration of phases following a deactivating switch (Figure 4). Observing the duration distribution of such phases (Figures 4A–4C), we identified promoter-specific variations in response to treatment. While the short *hGH* promoter-produced periods remain unchanged when treated, the durations produced by the larger *hGH* promoter are significantly altered. The median phase duration of both the −3,348/+1 bp *hGH* and *hPrl* promoter is increased by HDAC inhibition. Interestingly, all three promoters produce an absolute minimum deactivated phase duration of 3 hr when unstimulated in BSA medium, suggesting a state during which transcription cannot be activated (Figure S4). For the larger *hGH* and *hPrl* promoter constructs, this period is reduced upon removal of HDAC activity. To statistically quantify this observation we developed a three-state model of transcription, refractory model (Figure 4D). This model infers the duration of two independent states (“off” and “primed”), which comprise the duration of the phase following a deactivating switch (Figures 4A–4C). Statistical modeling identified significant reductions in the duration of the refractory state for each promoter following specific treatments (Figures 4E–4G). Most notably, the median refractory phase of the shorter *hGH* promoter is reduced from 4.3 hr (in BSA) to 1.9 hr with Fsk treatment, while the larger

hGH promoter requires HDAC inhibition to reduce this period from 3.2 hr (in BSA) to 1.9 hr with a combination of Fsk and TSA. Therefore, we suggest that a property of the larger fragments is the increased regulation of transcriptional burst duration and frequency through the increased degree of chromatin remodeling. Interestingly, we also observe a system memory related to the duration of phases of increased activity, with a minimum period of sustained activation of approximately 50 min required for each promoter (Figure S4).

DISCUSSION

The timing of gene expression in individual cells has emerged as a fundamental aspect of physiological regulation (Chubb et al., 2006; Piras et al., 2014; Featherstone et al., 2016). Recent work describing transcription dynamics in living cells and tissues has shown that at all levels from signaling to protein synthesis, gene expression control is far more dynamic than was once thought (Harper et al., 2011; Molina et al., 2013). The general view has been that these dynamics arise from stochastic switching between on- and off-states of transcription. The observation of the importance of a refractory phase in the pulsatility of gene expression (Harper et al., 2011; Suter et al., 2011; Cesbron et al., 2015; Zoller et al., 2015) has suggested the idea that the timing may involve a key period of chromatin remodeling that determines characteristic transcription dynamics for a given gene. Here, we show the non-intuitive result that there is a consistent asymmetry between the probability of switching between activation and deactivation of transcription for different promoters, with different integration sites. While the occasional stepwise increase in transcriptional rates is possible, we predominantly observe a single, all-or-nothing activating switch followed by several steps of graded reductions of decreasing amplitude. These results arise from unbiased statistical analysis of time-lapse reporter gene expression. The picture that emerges is that genes tend to achieve the highest transcriptional state of the current cycle within a single switch, but then switch down through a series of different levels of transcription with the first switch often being the largest (Figure 2). Thus in a given period we observe more down-switches than up-switches, even in a population of cells that are maintaining a stable level of gene expression. This phenomenon was observed for two different *hGH* promoter constructs (Figure S1), and also for the prolactin promoter. Although these genes are evolutionarily related and are both regulated by the Pit-1 transcription factor, they display different structural characteristics and organization, and cell-type and development-specific expression.

Transitions between binary and graded processes may allow for the tuning of transcriptional output according to the initial activating stimulus. The binary-like activating switch is likely to be a function of several cumulative transcription-initiating interactions breaching a threshold of activation. Above this threshold, the increasing promoter and transcription factor interaction may increase the probability and opportunity of establishing a stable transcription complex, inhibiting further graded increases and recruiting polymerase II (Pol II) to its current maximal capability. Following activation, these promoter interactions may degrade and dissociate at varying rates, along with Pol II pausing, which will therefore gradually reduce the overall rate of transcription in a stepwise manner until a level of activity is met that reflects the new cell state (Saccani et al., 2004; Seila et al., 2009; Hammar et al., 2014; Jonkers and Lis, 2015). Larger promoter fragments driving the luciferase reporter appeared to direct an increased number of down-switches during the stepwise decay, likely due to more extensive and maintained chromatin status and a greater number of promoter-protein interactions.

The stochastic bursting of transcriptional activity is believed to be a general phenomenon, with temporal dynamics being highly promoter specific (Raj and Oudenaarden, 2009; Suter et al., 2011; Coulon et al., 2013), and individual transcription events for specific mRNAs occurring on a time scale of minutes (Muramoto et al., 2012). We show here that longer-scale maintenance of chromatin status is associated with defined characteristics of timing in the transcriptional cycles that we observe. Gene-specific phases of transcription and refractory periods are thought to contribute to the cyclic bursts, and their variability results in heterogeneous transcriptional states across a population of cells or tissue (Harper et al., 2010; Featherstone et al., 2011, 2016). In the present study, removal of chromatin-remodeling processes through HDAC inhibition removes this heterogeneity and alters the defined bursting pattern. Evidence here suggests that simpler promoters may be more subject to fluctuations in extrinsic noise through modification of transcriptional phases upon stimulation of signaling pathways. It is important to note that differing promoters with different architecture and complexity here display essentially the same patterns of activation and inactivation. In the same way that bursting behavior has been seen as a common feature for multiple regulated promoters (Suter et al., 2011), it may therefore be true that binary activation and graded inactivation are a common transcriptional phenotype. The potential transcription factor binding sites in the two GH promoters are shown in Figure S1, but the exact role of specific response elements and factor binding sites remains to be evaluated in detail.

Unbiased variable-rate modeling provides a robust inference of the underlying dynamics of transcription. This modeling approach has previously been evaluated using biological and synthetic data (Hey et al., 2015). Here we have tested its inferences regarding mRNA molecule number by direct assay using smFISH. Our statistical analysis has shown that transcription is likely to occupy a distribution of active rates. Although we still categorize transcription into discrete phases, these phases display a spectrum of rates (Molina et al., 2013; Featherstone et al., 2016; Corrigan et al., 2016). In addition to the previously identified gene-specific transcriptional burst frequency, amplitude, and duration, we also observe promoter-specific graded switching patterns. Greater promoter complexity is likely to support a rich variety of epigenetic control mechanisms to generate accurate regulation across different time domains in cells and tissues.

STAR★METHODS

Detailed methods are provided in the online version of this paper and include the following:

- KEY RESOURCES TABLE
- CONTACT FOR REAGENT AND RESOURCE SHARING

- EXPERIMENTAL MODEL AND SUBJECT DETAILS
  - Cell Lines
- METHOD DETAILS
  - Plasmid Preparation and Transfection
  - Copy Number Validation
  - Genomic DNA Extraction
  - qRT-PCR
  - Real-Time Luminescence Imaging
  - Single Molecule RNA-*In Situ* Hybridisation
  - Quantification and Statistical Analysis
  - Protein and mRNA Half-Life Estimation
- DATA AND SOFTWARE AVAILABILITY

SUPPLEMENTAL INFORMATION

Supplemental Information includes four figures and three tables and can be found with this article online at https://doi.org/10.1016/j.cels.2017.10.013.

AUTHOR CONTRIBUTIONS

L.S.S.D., H.M., M.R.H.W., and J.R.E.D. designed research; L.S.S.D., C.V.H., and P.J.D. performed research; H.M., C.V.H., K.H., D.G.S., A.M., and K.F. contributed new reagents/analytic tools; L.S.S.D. and H.M. analyzed data; D.A.R., B.F., M.R.H.W., and J.R.E.D. supervised research; L.S.S.D., M.R.H.W., and J.R.E.D. wrote the paper.

ACKNOWLEDGMENTS

We thank Jonathan Chubb and Prof. Andrew Sharrocks for helpful critical discussion of the manuscript, and Paul Le Tissier for discussions throughout this project. Hamamatsu Photonics and Carl Zeiss Limited provided technical support to the Systems Microscopy Centre. The Delta Vision microscope used in the study was purchased with grants from BBSRC, Wellcome, and the University of Manchester Strategic Fund. L.S.S.D. was supported by the Biotechnology and Biological Sciences Research Council (BB/F01760X/1). We acknowledge support from the Wellcome Trust (program grant 091688) and the Medical Research Council (MR/K015885/1 and MR/M013170/1). K.H. was supported by the Engineering and Physical Sciences Research Council (ASTAA1112.KXH). The Faculty of Biology, Medicine & Health is supported by the Manchester Academic Health Sciences Centre (MAHSC). H.M. and D.A.R. were supported by BBSRC sLola BB/K003097/1 and by funding from the European Union Seventh Framework Programme (FP7/2007-2013) under grant agreement no. 305564.

Received: October 26, 2016
Revised: August 4, 2017
Accepted: October 18, 2017
Published: November 15, 2017

REFERENCES

Alonso, M., Segura, C., Dieguez, C., and Perez-Fernandez, R. (1998). High-affinity binding sites to the vitamin D receptor DNA binding domain in the human growth hormone promoter. Biochem. Biophys. Res. Commun. *247*, 882–887.

Becskei, A., Kaufmann, B.B., and van Oudenaarden, A. (2005). Contributions of low molecule number and chromosomal positioning to stochastic gene expression. Nat. Genet. *37*, 937–944.

Biggar, S.R., and Crabtree, G.R. (2001). Cell signaling can direct either binary or graded transcriptional responses. EMBO J. *20*, 3167–3176.

Bintu, L., Yong, J., Antebi, Y.E., McCue, K., Kazuki, Y., Uno, N., Oshimura, M., and Elowitz, M.B. (2016). Dynamics of epigenetic regulation at the single-cell level. Science *351*, 720–724.

Bodner, M., Castriilo, J.-L., Theill, L.E., Deerinck, T., Ellisman, M., and Karin, M. (1988). The pituitary-specific transcription factor GHF-1 is a homeobox-containing protein. Cell *55*, 505–518.

Carey, L.B., van Dijk, D., Sloot, P.M.A., Kaandorp, J.A., and Segal, E. (2013). Promoter sequence determines the relationship between expression level and noise. PLoS Biol. *11*, e1001528.

Cesbron, F., Oehler, M., Ha, N., Sancar, G., and Brunner, M. (2015). Transcriptional refractoriness is dependent on core promoter architecture. Nat. Commun. *6*, 6753.

Chubb, J.R., Trcek, T., Shenoy, S.M., and Singer, R.H. (2006). Transcriptional pulsing of a developmental gene. Curr. Biol. *16*, 1018–1025.

Cohen, L.E., Hashimoto, Y., Zanger, K., Wondisford, F., and Radovick, S. (1999). CREB-independent regulation by CBP is a novel mechanism of human growth hormone gene expression. J. Clin. Invest. *104*, 1123–1130.

Corrigan, A.M., and Chubb, J.R. (2014). Regulation of transcriptional bursting by a naturally oscillating signal. Curr. Biol. *24*, 205–211.

Corrigan, A.M., Tunnacliffe, E., Cannon, D., and Chubb, J.R. (2016). A continuum model of transcriptional bursting. Elife *5*, e13051.

Coulon, A., Chow, C.C., Singer, R.H., and Larson, D.R. (2013). Eukaryotic transcriptional dynamics: from single molecules to cell populations. Nat. Rev. Genet. *14*, 572–584.

Dadiani, M., van Dijk, D., Segal, B., Field, Y., Ben-Artzi, G., Raveh-Sadka, T., Levo, M., Kaplow, I., Weinberger, A., and Segal, E. (2013). Two DNA-encoded strategies for increasing expression with opposing effects on promoter dynamics and transcriptional noise. Genome Res. *23*, 966–976.

Edelstein, A., Tsuchida, M., Amodai, N., Pinkard, H., Vale, R., and Stuurman, N. (2014). Advanced methods of microscope control using μManager software. J. Biol. Methods *1*, e11.

Elowitz, M.B., Levine, A.J., Siggia, E.D., and Swain, P.S. (2002). Stochastic gene expression in a single cell. Science *297*, 1183–1186.

Featherstone, K., Harper, C.V., Mcnamara, A., Semprini, S., Spiller, D.G., Mcneilly, J., Mcneilly, A.S., Mullins, J.J., White, M.R.H., and Davis, J.R.E. (2011). Pulsatile patterns of pituitary hormone gene expression change during development. J. Cell Sci. *124*, 3484–3491.

Featherstone, K., Hey, K., Momiji, H., McNamara, A.V., Patist, A.L., Woodburn, J., Spiller, D.G., Christian, H.C., McNeilly, A.S., Mullins, J.J., et al. (2016). Spatially coordinated dynamic gene transcription in living pituitary tissue. Elife *5*, e08494.

Giorgetti, L., Siggers, T., Tiana, G., Caprara, G., Notarbartolo, S., Corona, T., Pasparakis, M., Milani, P., Bulyk, M.L., and Natoli, G. (2010). Noncooperative interactions between transcription factors and clustered DNA binding sites enable graded transcriptional responses to environmental inputs. Mol. Cell *37*, 418–428.

Green, P.J. (1995). Reversible jump Markov chain Monte Carlo computation and Bayesian model determination. Biometrika *82*, 711–732.

Hammar, P., Wallden, M., Fange, D., Persson, F., Baltekin, O., Ullman, G., Leroy, P., and Elf, J. (2014). Direct measurement of transcription factor dissociation excludes a simple operator occupancy model for gene regulation. Nat. Genet. *46*, 405–408.

Harper, C.V., Featherstone, K., Semprini, S., Friedrichsen, S., McNeilly, J., Paszek, P., Spiller, D.G., McNeilly, A.S., Mullins, J.J., Davis, J.R.E., and White, M.R.H. (2010). Dynamic organisation of prolactin gene expression in living pituitary tissue. J. Cell Sci. *123*, 424–430.

Harper, C.V., Finkenstädt, B., Woodcock, D.J., Friedrichsen, S., Semprini, S., Ashall, L., Spiller, D.G., Mullins, J.J., Rand, D.A., Davis, J.R.E., and White, M.R.H. (2011). Dynamic analysis of stochastic transcription cycles. PLoS Biol. *9*, e1000607.

Hey, K.L., Momiji, H., Featherstone, K., Davis, J.R.E., White, M.R.H., Rand, D.A., and Finkenstädt, B. (2015). A stochastic transcriptional switch model for single cell imaging data. Biostatistics *16*, 655–669.

Ho, Y., Cooke, N.E., and Liebhaber, S.A. (2015). An auto-regulatory pathway establishes the definitive chromatin conformation at the Pit-1 locus. Mol. Cell. Biol. *35*, 1523–1532.

Ho, Y., Elefant, F., Cooke, N., and Liebhaber, S. (2002). A defined locus control region determinant links chromatin domain acetylation with long-range gene activation. Mol. Cell *9*, 291–302.

Ho, Y., Liebhaber, S.A., and Cooke, N.E. (2011). The role of the hGH locus control region in somatotrope restriction of hGH-N gene expression. Mol. Endocrinol. *25*, 877–884.

Hocine, S., Vera, M., Zenklusen, D., and Singer, R.H. (2015). Promoter-autonomous functioning in a controlled environment using single molecule FISH. Sci. Rep. *5*, 9934.

Jenkins, D.J., Finkenstädt, B., and Rand, D.A. (2013). A temporal switch model for estimating transcriptional activity in gene expression. Bioinformatics *29*, 1158–1165.

Jin, Y., Surahbi, R.M., Fresnoza, A., Lytras, A., and Cattini, P.A. (1999). A role for A/T rich sequences and Pit-1/GHF-1 in a distal enhancer located in the human growth hormone locus control region with preferential pituitary activity in culture and transgenic mice. Mol. Endocrinol. *13*, 1249–1266.

Jones, B.K., Monks, B.R., Liebhaber, S.A., and Cooke, N.E. (1995). The human growth hormone gene is regulated by a multicomponent locus control region. Mol. Cell. Biol. *15*, 7010–7021.

Jonkers, I., and Lis, J.T. (2015). Getting up to speed with transcription elongation by RNA polymerase II. Nat. Rev. Mol. Cell. Biol. *16*, 167–177.

Kent, W.J., Sugnet, C.W., Furey, T.S., Roskin, K.M., Pringle, T.H., Zahler, A.M., and Haussler, D. (2002). The human genome browser at UCSC. Genome Res. *12*, 996–1006.

Larson, D.R., Zenklusen, D., Wu, B., Chao, J.A., and Singer, R.H. (2011). Real-time observation of transcription initiation and elongation on an endogenous yeast gene. Science *332*, 475–478.

Lipkin, S.M., Näär, A.M., Kalla, K.A., Sack, R.A., and Rosenfeld, M.G. (1993). Identification of a novel zinc finger protein binding a conserved element critical for Pit-1-dependent growth hormone gene expression. Genes Dev. *7*, 1674–1687.

Matys, V., Kel-Margoulis, O.V., Fricke, E., Liebich, I., Land, S., Barre-Dirrie, A., Reuter, I., Chekmenev, D., Krull, M., Hornischer, K., et al. (2006). TRANSFAC and its module TRANSCompel: transcriptional gene regulation in eukaryotes. Nucleic Acid Res. *34*, D108–D110.

McNamara, A.V., Adamson, A.D., Dunham, L.S.S., Semprini, S., Spiller, D.G., McNeilly, A.S., Mullins, J.J., Davis, J.R.E., and White, M.R.H. (2016). Role of estrogen response element in the human prolactin gene: transcriptional response and timing. Mol. Endocrinol. *30*, 189–200.

Molina, N., Suter, D.M., Cannavo, R., Zoller, B., Gotic, I., and Naef, F. (2013). Stimulus-induced modulation of transcriptional bursting in a single mammalian gene. Proc. Natl. Acad. Sci. USA *110*, 20563–20568.

Mueller, F., Senecal, A., Tantale, K., Marie-Nelly, H., Ly, N., Collin, O., Basyuk, E., Bertrand, E., Darzacq, X., and Zimmer, C. (2013). FISH-quant: automatic counting of transcripts in 3D FISH images. Nat. Methods *10*, 277–278.

Muramoto, T., Cannon, D., Gierliński, M., Corrigan, A., Barton, G.J., and Chubb, J.R. (2012). Live imaging of nascent RNA dynamics reveals distinct types of transcriptional pulse regulation. Proc. Natl. Acad. Sci. USA *109*, 7350–7355.

Neuert, G., Munsky, B., Tan, R.Z., Teytelman, L., Khammash, M., and van Oudenaarden, A. (2013). Systematic identification of signal-activated stochastic gene regulation. Science *339*, 584–587.

Niall, H.D., Hogan, M.L., Sauer, R., Rosenblum, I.Y., and Greenwood, F.C. (1971). Sequences of pituitary and placental lactogenic and growth hormones: evolution from a primordial peptide by gene reduplication. Proc. Natl. Acad. Sci. USA *68*, 866–869.

Noordermeer, D., de Wit, E., Klous, P., van de Werken, H., Simonis, M., Lopez-Jones, M., Eussen, B., de Klein, A., Singer, R.H., and de Laat, W. (2011). Variegated gene expression caused by cell-specific long-range DNA interactions. Nat. Cell Biol. *13*, 944–951.

Norris, A.J., Stirland, J.A., McFerran, D.W., Seymour, Z.C., Spiller, D.G., Loudon, A.S.I., White, M.R.H., and Davis, J.R.E. (2003). Dynamic patterns of growth hormone gene transcription in individual living pituitary cells. Mol. Endocrinol. *17*, 193–202.

Ochab-Marcinek, A., and Tabaka, M. (2015). Transcriptional leakage versus noise: a simple mechanism of conversion between binary and graded response in autoregulated genes. Phys. Rev. E Stat. Nonlin. Soft Matter Phys. *91*, 012704.

Pawlak, A., Lahuna, O., Bulle, F., Suzuki, A., Ferry, N., Siegrist, S., Chikhi, N., Chobert, M.N., Guellaen, G., and Laperche, Y. (1988). gamma-Glutamyl transpeptidase: a single copy gene in the rat and a multigene family in the human genome. J. Biol. Chem. *263*, 9913–9916.

Piras, V., Tomita, M., and Selvarajoo, K. (2014). Transcriptome-wide variability in single embryonic development cells. Sci. Rep. *4*, 7137.

Raj, A., and Oudenaarden, A.V. (2009). Single-molecule approaches to stochastic gene expression. Annu. Rev. Biophys. *38*, 255–270.

Raj, A., Peskin, C.S., Tranchina, D., Vargas, D.Y., and Tyagi, S. (2006). Stochastic mRNA synthesis in mammalian cells. PLoS Biol. *4* (10), e309.

Saccani, S., Marazzi, I., Beg, A.A., and Natoli, G. (2004). Degradation of promoter-bound p65/RelA Is essential for the prompt termination of the nuclear factor κB response. J. Exp. Med. *200*, 107–113.

Schneider, C., Rasband, W., and Eliceri, K. (2012). NIH image to ImageJ: 25 years of image analysis. Nat. Methods *9*, 671–675.

Seila, A.C., Core, L.J., Lis, J.T., and Sharp, P.A. (2009). Divergent transcription: a new feature of active promoters. Cell Cycle *8*, 2557–2564.

Semprini, S., Friedrichsen, S., Harper, C.V., McNeilly, J.R., Adamson, A.D., Spiller, D.G., Kotelevtseva, N., Brooker, G., Brownstein, D.G., McNeilly, A.S., et al. (2009). Real-time visualization of human prolactin alternate promoter usage in vivo using a double-transgenic rat model. Mol. Endocrinol. *23*, 529–538.

Sepúlveda, L.A., Xu, H., Zhang, J., Wang, M., and Golding, I. (2016). Measurement of gene regulation in individual cells reveals rapid switching between promoter states. Science *351*, 1218–1222.

Shewchuk, B.M., Ho, Y., Liebhaber, S.A., and Cooke, N.E. (2006). A single base difference between Pit-1 binding sites at the hGH promoter and locus control region specifies distinct Pit-1 conformations and functions. Mol. Cell. Biol. *26*, 6535–6546.

Shewchuk, B.M., Liebhaber, S.A., and Cooke, N.E. (2002). Specification of unique Pit-1 activity in the hGH locus control region. Proc. Natl. Acad. Sci. USA *99*, 11784–11789.

Sorre, B., Warmflash, A., Brivanlou, Ali H., and Siggia, Eric D. (2014). Encoding of temporal signals by the TGF-β pathway and implications for embryonic patterning. Dev. Cell *30*, 334–342.

Suter, D.M., Molina, N., Gatfield, D., Schneider, K., Schibler, U., and Naef, F. (2011). Mammalian genes are transcribed with widely different bursting kinetics. Science *332*, 472–474.

Takasuka, N., White, M.R.H., Wood, C.D., Robertson, W.R., and Davis, J.R.E. (1998). Dynamic changes in prolactin promoter activation in individual living lactotrophic cells. Endocrinology *139*, 1361–1368.

Tashjian, A., Yasumura, Y., Levine, L., Sato, G., and Parker, M. (1968). Establishment of clonal strains of rat pituitary tumor cells that secrete growth hormone. Endocrinology *82*, 342–352.

Zenklusen, D., Larson, D.R., and Singer, R.H. (2008). Single-RNA counting reveals alternative modes of gene expression in yeast. Nat. Struct. Mol. Biol. *15*, 1263–1271.

Zhang, J., and Zhou, T. (2014). Promoter-mediated transcriptional dynamics. Biophys. J. *106*, 479–488.

Zoller, B., Nicolas, D., Molina, N., and Naef, F. (2015). Structure of silent transcription intervals and noise characteristics of mammalian genes. Mol. Syst. Biol. *11*, 823.

STAR★METHODS

KEY RESOURCES TABLE

REAGENT or RESOURCE	SOURCE	IDENTIFIER
Bacterial and Virus Strains		
DH5a Competent Cells	Invitrogen	18265017
Chemicals, Peptides, and Recombinant Proteins		
Forskolin	Sigma-Aldrich	F3917
Trichostatin A	Sigma-Aldrich	T1952
Cycloheximide	Sigma-Aldrich	C104450
Actinomycin D	Sigma-Aldrich	01815
Critical Commercial Assays		
FuGENE Transfection reagent	Promega	E2311
Copy number assay kit	ThermoFisher	4442487
PureLink Genomic DNA kit	ThermoFisher	K182001
Stellaris FISH	Biosearch Technologies	VSMF-1007-5
Miniprep	Qiagen	27104
Maxiprep	Qiagen	10023
Deposited Data		
Raw data	This paper	N/A
Experimental Models: Cell Lines		
GH3	Tashjian et al., 1968	N/A
GH3/hGH3348	This paper	N/A
GH3/hGH840	This paper	N/A
GH3/hPrl	This paper	N/A
Oligonucleotides		
Copy number Taqman probe	FAM-AATTGCTCAACAGTATGGG-MGB	N/A
Copy number luciferase forward primer	CCGCGAACGACATTTATAATGA	N/A
Copy number luciferase reverse primer	CCACGGTAGGCTGCGAAA	N/A
GGT1 probe	Cy5-CCGAGAAGCAGCCACAGCCATACCT-BHQ2	N/A
GGT1 forward primer	CCACCCCTTCCCTACTCCTAC	N/A
GGT1 reverse primer	GGCCACAGAGCTGGTTGTC	N/A
Recombinant DNA		
pGL3-hGH3348/+1-luciferase	This paper	hGH-3348/+1bp
pGL3-hGH -840/+1-luciferase	This paper	hGH-840/+1bp
pGL3-hGH496/+1-luciferase	Norris et al., 2003	hGH-496/+1bp
Cosmid K2B	Jones et al., 1995	N/A
pGL3-5kbhPrl- luciferase	Takasuka et al., 1998	hPrl
pGL3-basic	Promega	E1751
pVITRO2-hyg-mcs	Invivogen	pvitro1-mcs
Software and Algorithms		
Micro-Manager (Version 1.4)	Edelstein et al., 2014	https://micro-manager.org/wiki/Micro-Manager_Version_Archive
UCSC Genome Browser	Kent et al., 2002	https://genome.ucsc.edu/
TRANSFAC 7.0 Public 2005	Matys et al., 2006	http://gene-regulation.com/pub/databases.html
AQM6 Image Analysis Software (Kinetic)	Andor	

(*Continued on next page*)

Continued		
REAGENT or RESOURCE	SOURCE	IDENTIFIER
Copy Caller Software v2.1	Applied Biosystems	https://www.thermofisher.com/it/en/home/technical-resources/software-downloads/copycaller-software.html
DeltaVision Microscopy Imaging Systems	General Electric	Softworx Suite 2.0
ImageJ	Schneider et al., 2012	https://imagej.nih.gov/ij/download.html
MATLAB R2015b (version 8.6)	MathWorks	N/A
Stochastic Switch Model	Hey et al., 2015	SSM
Refractory Model	This paper	N/A

CONTACT FOR REAGENT AND RESOURCE SHARING

Further information and requests for resources and reagents should be directed to and will be fulfilled by the Lead Contact Julian Davis (julian.davis@manchester.ac.uk).

EXPERIMENTAL MODEL AND SUBJECT DETAILS

Cell Lines

Female rat pituitary GH3 cells were used as a model in this study (Tashjian et al., 1968). These cells require growth in phenol-red free DMEM with pyruvate (Gibco) supplemented with 1% glutamine, and 10% fetal calf serum (Gibco). All imaging was performed in serum-starved conditions in phenol-red free DMEM with pyruvate, 1% glutamine, and 0.25% bovine serum albumin (Gibco). Cells were maintained at 37°C and 5% CO_2.

METHOD DETAILS

Plasmid Preparation and Transfection

Human growth hormone promoter-luciferase constructs were generated via amplification from the cosmid K2B (Jones et al., 1995). The amplified fragments were digested with *Sac1* and ligated into the *Sac1* digested pGL3-basic plasmid (Promega) 5' to the luciferase reporter gene coding sequence. Generation of the 5kb prolactin-luciferase construct and cell line has been previously described (Takasuka et al., 1998). 10μg of either plasmid was isolated via miniprep (Qiagen) and was mixed with 3ug pVITRO2-hyg-mcs in 1ml of 150mM NaCl to allow for co-transfection into 1 x 10^5 GH3 cells using FuGENE transfection reagent (Promega). Media was changed every 3 days post transfection, and was supplemented with 500μg/ml hygromycin selection antibiotic. Positively transformed cells formed colonies after 2-3 weeks and were ring cloned into individual wells of a 48 well plate and cultured. The clones were screened for luciferase expression and response to stimuli (5nM forskolin, 100nM dexamethasone, and 50μM T_3), prior to establishing gene copy number.

Copy Number Validation

The copy number of GH3 transfected luciferase constructs was quantified using a customisable copy number assay kit (Life Technologies), comparing qRT-PCR amplification of the luciferase gene with known controls.

Genomic DNA Extraction

The PureLink Genomic DNA kit (Life Technologies) was used to extract all genomic DNA (gDNA) from the transformed GH3 cell lines following the manufacturer's instructions. gDNA concentration and purity was quantified using a Nanodrop 2000 UV-Vis spectrophotomer (Thermo Scientific). The absorbance was measured between 260 and 280nm. All gDNA were of sufficient purity with a A_{260}/A_{280} ration between 1.9-2.1.

qRT-PCR

The gDNA was amplified using the copy number assay kit (Life Technologies). 20ng of gDNA was mixed with manufacturer instructed amounts of TaqMan genotyping master mix, copy number assay, copy number reference assay and nuclease-free water. The copy number assay contains a TaqMan probe and quencher directed to the luciferase using customised luciferase oligonucleotides (Forward- CCGCGAACGACATTTATAATGA; Reverse- CCACGGTAGGCTGCGAAA; Probe- FAM-AATTGCTCAACAGTATGGG-MGB) (Semprini et al., 2009). Amplification of the luciferase target was compared directly to the amplification of the GGT1

housekeeping gene using primers and a probe previously described by Pawlak et al. (1988) (Fwd- CCACCCCTTCCCTACTCCTAC; Rev- GGCCACAGAGCTGGTTGTC; Probe- Cy5-CCGAGAAGCAGCCACAGCCATACCT-BHQ2). qRT-PCR was performed using a StepOnePlus (Applied Biosystems) with cycling parameters as follows: initial denaturation at 95°C for 10 min; 40 cycles of 95°C 15 secs, 60°C 60 secs; hold at 72°C for 5 min.

Real-Time Luminescence Imaging

Image Acquisition

1 x 10^5 transformed GH3 cells were plated onto a CELLview 35mm glass-bottomed cell culture dish in FCS-containing media. 24h prior to imaging cells were washed with PBS and serum starved in media containing 1mM luciferin. Application of stimuli was performed immediately prior to transfer to the stage of a Zeiss Axiovert 200 equipped with an XL incubator to maintain the cells at 37°C, 5% CO_2. Luminescence images were obtained using a Fluar 10x, 0.5NA objective (Zeiss) and captured using an ImagEM EM-CCD cooled camera (Hamamatsu photonics). Images were integrated over a 15m period using a 1 x 1 binning and acquired using Micro-Manager software (Version 1.4). In treated conditions, forskolin (50μM) and trichostatin A (50nM) were added immediately prior to imaging.

Protein and mRNA Inhibition

For the back-calculation from observed luminescence to the estimated transcriptional dynamics we required the calculation of mRNA and protein degradation rates. Whilst both hGH-luciferase constructs have identical 3' UTRs, their respective genome insertion sites may vary and therefore affect post-translational modification and mRNA and protein half-lives. We imaged single-cell bioluminescence of transformed GH3 cells when stimulated with 5nM forskolin followed by the application of cycloheximide (10μg/ml) or actinomycin D (3μg/ml) (Sigma) to block translation and transcription, respectively.

Single Molecule RNA-*In Situ* Hybridisation

Sample Preparation

4 x 10^4 transformed GH3 cells were plated in FCS-containing media onto glass coverslips pre-treated with poly-L-lysine. Cells were cultured for two days, treated with 5μM forskolin or left untreated, and fixed four hours later. Coverslips were fixed then hybridised with the Stellaris FISH probe set against firefly luciferase conjugated with Quasar 670 dye (VSMF-1007-5, Biosearch Technologies), using the manufacturer's protocol for adherent cells.

Image Acquisition

Images were obtained using a Delta Vision Core restoration microscope (Applied Precision) using a 60x/NA 1.42 Plan Apo objective and Sedat Quad filter set (Chroma Technology), and collected using a Coolsnap HQ2 camera (photometrics)(Mueller et al., 2013).

Quantification and Statistical Analysis

Bioinformatic Promoter Analysis

hGH promoter sequence was obtained using the UCSC Genome Browser (http://genome.ucsc.edu/), locating the hGH start site and exporting the upstream promoter sequence. Transfac analysis was performed on the -3348bp of promoter sequence using weight limits: matrix = 0.9, core = 0.95 to identify likely transcription factor binding sites (TRANSFAC 7.0 Public 2005).

Analysis of Image Data

Individual cells were tracked using AQM6 image analysis software (Kinetic). Regions of interest were drawn around each individual cell, and mean intensity data collected. Monadic noise between individual frames was removed, and background noise was subtracted from the luminescence signal. Analysis of an individual cell ceased at the point of cell division.

qRT-PCR Analysis

Relative fluorescence was then analysed using Copy Caller Software (ThermoFisher) generating a relative quantification value for number of gene copies within each clone.

FISH Data Processing

Raw images were deconvolved using the Softworx software then converted to .tif stacks using a custom ImageJ script. Transcript counts for each cell were determined using FISH-quant (Jin et al., 1999).

Correlation Coefficient Calculation

To analyse the temporal correlation of luminescence dynamics in response to specific treatments we calculated the correlation coefficient for sequential 1h pooled periods. The correlation of luminescence throughout an hour period was calculated between each and every single cell under a particular treatment. The median and distribution of these hour correlation coefficients were plotted for a 20h period demonstrating the degree of similarity between individual cells. The significance of each pool was compared using an unpaired t-test.

Stochastic Switch Model

To estimate switches between variable rates of transcription, we applied a previously developed stochastic switch model (Hey et al., 2015). This reversible jump Markov chain Monte Carlo algorithm estimates the temporal location of a significant change in transcriptional activity through the back-calculation of protein and mRNA degradation rates (which remain unchanged by stimulation, Table

S2). The below stochastic reaction network is incorporated within this model, allowing for the identification of luminescence above stochastic noise within the system:

$$\varnothing \xrightarrow{\beta(t)} \text{mRNA}$$

$$\text{mRNA} \xrightarrow{\delta_m} \varnothing$$

$$\text{mRNA} \xrightarrow{\alpha} \text{mRNA} + \text{Protein}$$

$$\text{Protein} \xrightarrow{\delta_p} \varnothing$$

The degradation rates of reporter mRNA and protein are denoted by δ_m and δ_p, respectively, whilst α denotes the rate of translation and β(t) denotes the time varying rate of transcription. Our transcription function is given by:

$$\beta(t) = \beta_i \textit{ for } t \in [s_{i-1}, s_i] \text{ for } i = 1, \ldots, K,$$

where K is the number of transcriptional switches, occurring at times $s_1, s_2, \ldots, s_K$ and $\beta_1, \beta_2, \ldots, \beta_K$ are the corresponding transcriptional rates. We impose no restriction to the form of the transcriptional levels but note that the conventional binary switch behaviour can be seen as a specific example where $\beta_i = \beta_{LOW}$ if the gene is inactive in the time period $[s_{i-1}, s_i]$ or $\beta_i = \beta_{HIGH}$ if the gene is active.

Assuming light intensity measurements are related to reporter protein levels by the equation,

$$Y(t) = \kappa P(t) + \varepsilon(t),$$

$$\varepsilon(t) \sim N(0, \sigma^2),$$

inference is performed through the linear noise approximation to the stochastic reaction network coupled with this measurement equation to obtain the posterior transcriptional function for each single cell. To ensure model identifiability, we impose informative prior distributions about the degradation parameters, obtained from independent half-life experiments. In addition, we specify a hierarchical framework over each dataset, as individual parameters are unlikely to change substantially.

In order to estimate both the number and positioning of transcriptional switches, we employ a reversible jump Markov Chain Monte Carlo (MCMC) algorithm (Green, 1995). Consequently, the posterior distribution consists of all possible transcriptional profiles. In order to extract the information regarding the estimated transcriptional dynamics, the posterior samples go through a post-processing procedure outlined below.

A parametric model is fitted to the marginal posterior switch distribution (as described by Jenkins et al., 2013). Specifically, a Gaussian mixture model is fitted to the marginal posterior distribution of the possible switch times.

All possible sub-models are extracted, to take into account the co-occurrence of switches. For example, if the marginal posterior has two possible switch positions, the sub-models will consist of a zero switch model, two mutually exclusive one switch models, and the two switch model. Counting the frequency with which each of the sub-models was sampled in the MCMC, we can associate a weight or probability to each sub-model.

Therefore, this post-processing procedure associates each single cell to a set of mutually exclusive transcriptional profiles. The analysis presented in the main paper has been calculated from the set of all possible transcriptional profiles, weighted by their probability of occurrence.

Protein and mRNA Half-Life Estimation

Half-life Calculation

We used these luminescence decay assays to first calculate the protein degradation rate (δ_P) using the function:

$$\frac{dP}{dt} = c_p - \delta_p P(t)$$

where c_p is a small non-negative constant.

This then allowed the estimation of the mRNA degradation rate (δ_M) from the transcription inhibition (actinomycin D) induced decay using the function,

$$\frac{dM}{dt} = c_M - \delta_M M(t)$$

where c_M is a small non-negative constant that is close to zero if transcription is fully inhibited.

Refractory Model

Statistical analyses of refractory (off) period modification were performed using a three-state model of transcription (Figure 3B). The parameter values were estimated by analysing the transcription profiles statistically inferred by SSM to real-time luciferase datasets. The maximum likelihood estimates of refractory and Off periods, T_1 and T_2, were obtained by fitting the sum of two exponential distributions to the durations after a down switch with the assumption of $T_1 < T_2$, while On period, T_0, was estimated by fitting an exponential distribution to the durations after an up switch. Both durations in the SSM results were analysed as right-censored ones. These period estimations are detailed below. Low and high rates, β_L and β_H, are the respective transcription rates after a down and up switch.

Maximum Likelihood Estimation of Refractory (T1), and Off (T2) Periods

In the following, capital T denotes a period parameter, while small t its realisation in the SSM-generated Markov chain, which represents the posterior distribution of the parameters. An inter-switch duration (t) is called complete if it is flanked by two switches, but right-censored if it is open-ended.

Pre-processing

In the continuous SSM, it is often the happening that a down or up switch is followed by the same-direction switch. To fit the SSM result into the current discrete on-off-primed model, such consecutive durations after same-direction switches are merged to form a single inter-switch duration.

Refractory and Off Periods Estimation

As Poisson processes, refractory and off periods, t_1 and t_2, follow respective exponential distributions, $t_1 \sim Exp(-\lambda_1\ t)$ and $t_2 \sim Exp(-\lambda_2\ t)$, where λ_1 and λ_2 denote respectively the inverse of T_1 and T_2. If $\lambda_1 \neq \lambda_1$ is assumed, the probability density function ($f_d(t_d)$) of the sum of those two random variables, $t_d = t_1 + t_2$ is given as follows.

$$f_d(t_d) = \lambda_1\ \lambda_2\ /\ (\lambda_2 - \lambda_1)\ (\exp(-\lambda_1\ t) - \exp(-\lambda_2\ t))$$

while its corresponding distribution function is given as follows.

$$F_d(t_d) = 1/(\lambda_2 - \lambda_1)\ \{\ \lambda_2\ (1 - \exp(-\lambda_1\ t)) - \lambda_1\ (1 - \exp(-\lambda_2\ t))\ \}$$

For given sets of complete and censored duration data, $\{t^{comp}{}_i\}$ and $\{t^{cens}{}_j\}$, a logarithmic likelihood is given as follows.

$$l(T_1, T_2) = \Sigma_i\ f_d(t^{comp}{}_i;\ T_1, T_2) + \Sigma_i\ (1 - F_d(t^{cens}{}_i;\ T_1, T_2))$$

With the assumption of $T_1 < T_2$, T_1 and T_2 are simultaneously estimated as argmax $l(T_1, T_2)$.

DATA AND SOFTWARE AVAILABILITY

Raw data of single cell luminescence profiles is available in Table S1.

Refractory Model. Matlab scripts can be freely accessed at Mendeley Data: https://doi.org/10.17632/wjyccvc2zc.2

These Matlab scripts will infer the duration of the refractory period from stochastic switch model-identified transcription periods.

Cell Systems, Volume *5*

Supplemental Information

Asymmetry between Activation and Deactivation during a Transcriptional Pulse

Lee S.S. Dunham, Hiroshi Momiji, Claire V. Harper, Polly J. Downton, Kirsty Hey, Anne McNamara, Karen Featherstone, David G. Spiller, David A. Rand, Bärbel Finkenstädt, Michael R.H. White, and Julian R.E. Davis

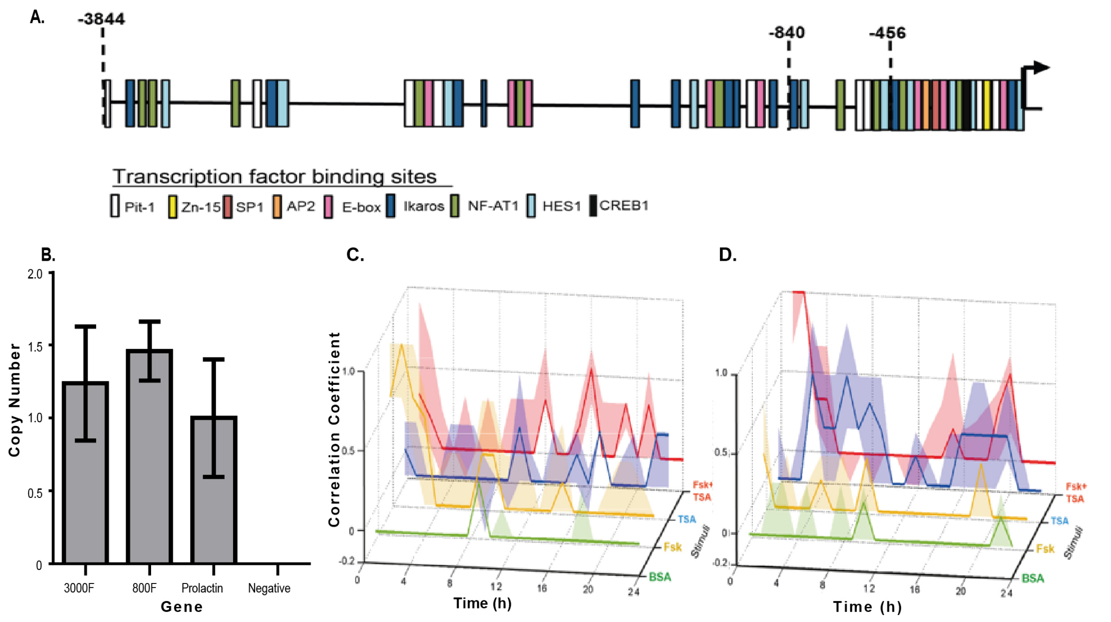

Figure S1 relating to Figure 1. A hGH promoter schematic of known transcription factor binding sites up to -456bp and bioinformatic estimated sites up to -3844bp. Binding site estimation performed with Transfac analysis weight limits: matrix = 0.9, core = 0.95. **B** Copy number validation of transformed GH3 cell lines, containing either the -840/+1bp or -3348/+1bp human growth hormone promoter driving the expression of the luciferase reporter gene. Each boxplot represents the mean copy number and standard deviation (*n = 3*). **C-D** Cell-cell luminescence pattern correlation in response to stimulation. Correlation coefficients were calculated of 1h pooled luminescence profiles for each cell pair combination within a population for a 20h observation period. The median correlation coefficient (bold) and the associated 95% confidence interval (shaded) demonstrates the coordination in luminescence activity in unstimulated (BSA) conditions (green), forskolin (Fsk) (yellow), trichostatin A (TSA) (blue), and Fsk + TSA (red).

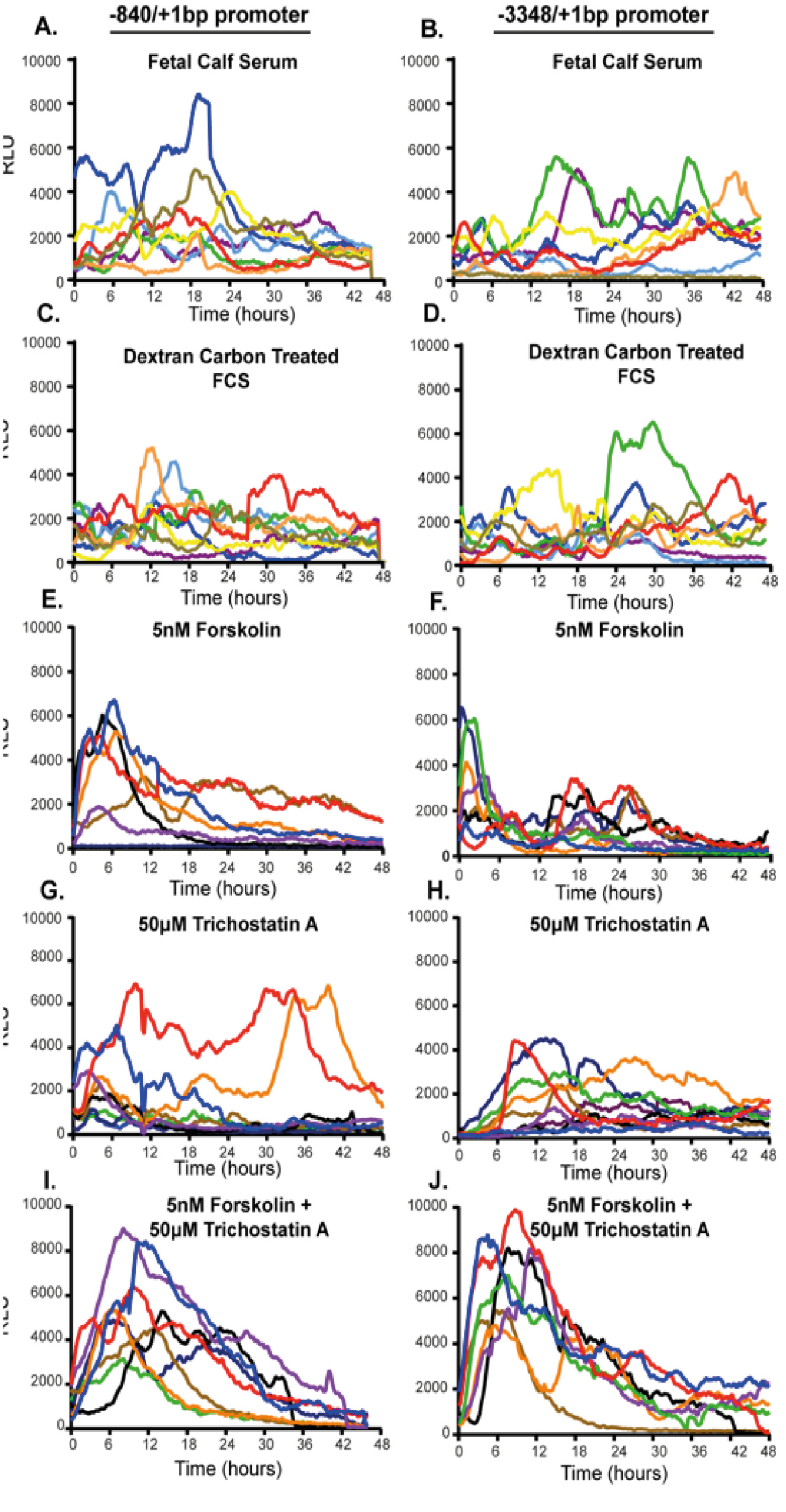

Figure S2 relating to Figure 1. A-J Representative single cell luminescence traces generated by -840/+1bp or -3348/+1bp human growth hormone promoter driven luciferase expression in GH3 cells, in response to serum and non-serum conditions, and stimulation with 5nM forskolin, 50μM trichostatin A, or a combination of both when maintained in BSA media. (-840/+1bp Fsk *n*=41, TSA *n*=33, Fsk+TSA *n*=46; -3348/+1bp Fsk *n*=59, TSA *n*=42, Fsk+TSA *n*=40).

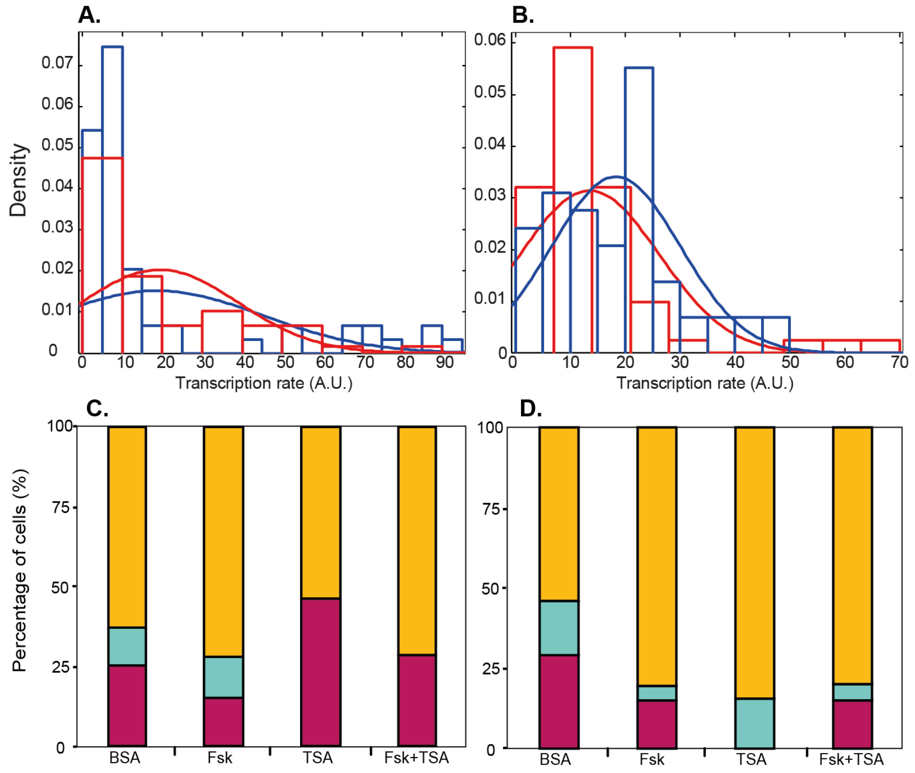

Figure S3 related to Figure 3. A-B Frequency distribution of stochastic switch model estimated transcription rates at the start (blue) and end (red) of the 48h observation period of the **A**. -840/+1bp and **B**. -3348/+1bp promoter-containing GH3 cells in BSA media. Kolmogorov-Smirnov test, $p>0.05$. **C-D** The percentage of cells in a population over a 48h period exhibiting either a binary, multi-step up, or multi-step down switch profile. When stimulated (Forskolin, trichostatin A, or combination) the percentage of cells containing either the **C**. hGH -840/+1bp or **D**. hGH -3348/+1bp promoter does not significantly change the switch profile they exhibit (ANOVA, $p > 0.05$).

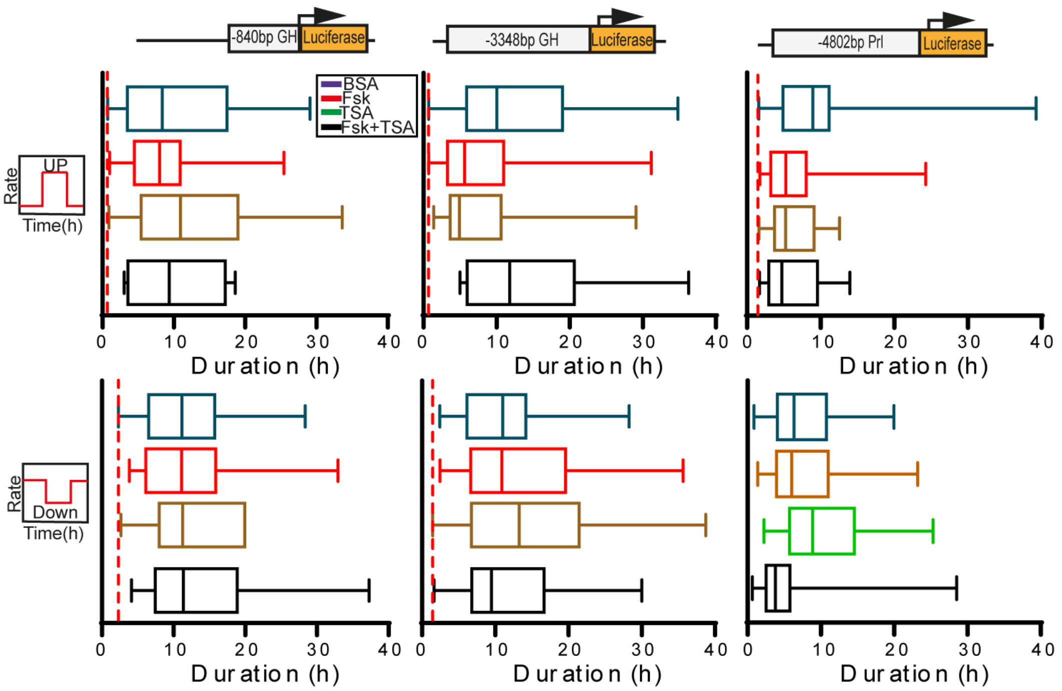

Figure S4 related to Figure 4. Boxplots demonstrating the median, interquartile range, and range of the stochastic switch model (SSM) estimated periods of transcription categorised as either Up or Down, depending upon the switch preceding each period for the -840/+1bp and -3348/+1bp hGH promoter constructs. Each boxplot represents the distribution of period durations in response to serum-starvation (BSA) (blue), forskolin (Fsk) (red), trichostatin A (TSA) (green), and a combination of Fsk and TSA (black).The dashed red line indicates the minimum estimated duration.

	BSA	Fsk	TSA	Fsk + TSA
Protein half-life	0.98 (±0.03) **58.8m**	0.98 (±0.02) **58.8m**	0.98 (±0.15) **58.8m**	1.11 (±0.08) **66.6m**
mRNA half-life	0.58 (±0.07) **34.6m**	0.6 (±0.01) **36m**	0.53(±0.01) **31.8m**	0.61(±0.02) **36m**

Table S2 related to Figure 2. Luciferase half-life used to determine protein and mRNA degradation rates following serum-starvation, or treatment with forskolin (Fsk) or trichostatin A (TSA). Half-life calculation (±SD) based on observed luminescence profile following treatment with cycloheximide (protein) or actinomycin D (mRNA half-life), and converted into minutes (**bold**).

		Promoter construct					
		**hGH -840/+1bp**		**hGH -3348/+1bp**		**hPrl -5kb**	
		Slope	R^2	*Slope*	R^2	*Slope*	R^2
**Consecutive switch directions**	*Up-Down*	-1.04 *(±0.04)*	*-0.95 *(±0.1)*	-1.08 *(±0.1)*	*0.99 *(±0.1)*	-0.96 *(±0.03)*	*0.94 *(±0.2)*
	Down-Down	1.76 *(±0.5)*	*0.60 *(±0.3)*	2.33 *(±0.9)*	*0.69 *(±0.1)*	1.92 *(±0.8)*	0.83 *(±0.1)*
	Up-Up	0.65 *(±1.7)*	-0.45 *(±0.9)*	-0.60 *(±0.3)*	0.39 *(±0.4)*	-0.33 *(±1.3)*	0.48 *(±0.4)*
	Down-Up	-0.73 *(±1.8)*	0.004 *(±1.9)*	0.15 *(±1.2)*	0.45 *(±0.7)*	0.26 *(±0.9)*	0.33 *(±0.5)*

Table S3 related to Figure 3. Mean (n=3) slope and R^2 values (±SD) calculated from the relationship between consecutive transcriptional switch amplitudes of -840/+1bp, -3348/+1bp human growth hormone and -5kb human prolactin promoters estimated by the stochastic switch model. *R^2>0.5.
